# Data and supplemental material of the paper “Effectiveness of digital-based interventions for children with mathematical learning difficulties: A meta-analysis”

**DOI:** 10.1016/j.dib.2020.105976

**Published:** 2020-07-03

**Authors:** Claudio Zandonella Callegher, Gianmarco Altoè

**Affiliations:** Department of Developmental Psychology and Socialization, University of Padova, Padova, Italy

**Keywords:** Dyscalculia, Mathematical disabilities, Mathematical difficulties, Digital-based tools, Media in education, Educational videogames, Intervention effects, Meta-analysis

## Abstract

Data and supplement material of the article “Effectiveness of digital-based interventions for children with mathematical learning difficulties: A meta-analysis” (Benavides-Varela et al.) [1] are presented. Data were collected from studies included in the meta-analysis to evaluate the effects of digital-based interventions for children with mathematical learning difficulties compared to control conditions in group-designed randomized controlled trials. Literature search, inclusion criteria and coding procedure are described. PRISMA flow-chart is reported to summarize the literature search and coding of all the relevant characteristics of the primary studies is made available. This allows other researchers to easily access to the information needed to evaluate the studies and to use these data in future meta-analyses. However, researchers are highly recommended to refer to the original papers in order to check studies suitability to their own criteria. Moreover, in the supplemental material all the information needed to reproduce the meta-analysis results is reported together with the R code syntax. Data and supplemental material are available online (https://osf.io/ajdnv/).

Specifications table

**Table:** 

**Subject**	Developmental and Educational Psychology
**Specific subject area**	Meta-analysis evaluating the effectiveness of digital-based interventions for children with mathematical learning difficulties.
**Type of data**	Tables Figures Supplemental Material with meta-analysis
**How data were acquired**	Data were collected from studies included in the meta-analysis. The aim of the meta-analysis was to evaluate the effects of digital-based interventions for children with mathematical learning difficulties compared to control conditions in group-designed randomized controlled trials. Primary studies acquired data about children's performance in different domain of mathematics. Each study used its own set of questionnaires and tests to evaluate mathematical performance of children with mathematical learning difficulties. Primary studies were selected through a literature search using the PRISMA protocol. The literature search was conducted until March 2019 by means of PsycINFO, Google Scholar, and Educational Resources Information Center (ERIC) databases. The following target terms were used to identify relevant studies: mathematics; dyscalculia; videogames; interventions; computer-assisted instruction; educational technology; mathematical learning; mathematics teaching; number sense; mathematics achievement; mathematical difficulties; randomized; controlled; control group; control condition. Eligible studies were selected according the following inclusion criteria: 1) Studies need to evaluate the effects of digital-based interventions for children with mathematical learning difficulties in a group-designed randomized controlled trial. 2) Studies need to report students’ performance in any domain of mathematics as their dependent variable in the pre- and post-intervention conditions. 3) Studies could have been conducted in any country, but only English-language articles published in peer-reviewed journals were included.
**Data format**	Raw Analyzed
**Parameters for data collection**	Selected studies differ according to the type of program used in the intervention (based on videogames or on tutoring and drilling strategies), intervention characteristics (length and topic of the intervention), control group characteristics (active or passive control group), school level of the participants (preschool, primary school, or high school), and outcomes measured to evaluate children's mathematical performance.
**Description of data collection**	Articles were screened for eligibility by three independent authors. A systematic coding form was used to record relevant information from each study. Selected studies were coded taking into account the characteristics of the intervention (length, type, and topic of the intervention), the characteristics of the control group (passive or active control group), the outcomes measured to evaluate children's mathematical performance, school level of the participants, sample size of the treatment and control group, and mean and standard deviation of the mathematical performance in the pre- and post-intervention conditions.
**Data source location**	
**Data accessibility**	Repository name: Effectiveness of digital-based interventions for children with mathematical learning difficulties: A meta-analysis Direct URL to data: https://osf.io/ajdnv/
**Related research article**	S. Benavides-Varela, C. Zandonella Callegher, B. Fagiolini, I. Leo, G. Altoè, D. Lucangeli, Effectiveness of digital-based interventions for children with mathematical learning difficulties: A meta-analysis. Comput. Educ. 157 (2020) 103953, https://doi.org/10.1016/j.compedu.2020.103953.

## Value of the data

• Why are these data useful? Literature search and coding of eligible studies are the most time-consuming part of a meta-analysis. These data clearly report and make available all the relevant characteristics of the primary studies selected for the meta-analysis. This allows other researchers to easily access to the information needed to evaluate these studies.
• Who can benefit from these data? Researchers who want to meta-analyze evidence about the effectiveness of digital-based interventions for children with mathematical learning difficulties can benefit from this data. This is an initial framework where more studies can be added to consider new evidence in the literature or evaluate the role of possible moderators. These data are also useful to check directly the results of the present meta-analysis or they can be used as a possible example for didactic purpose.
• How can these data be used for further insights and development of experiments? On the base of these data it is possible to plan new experiments aimed to evaluate the effectiveness of digital-based interventions for children with mathematical learning difficulties. An estimation of the “*plausible effect size*” can be computed from these data together with other information (i.e., other studies results or experts’ knowledge) and used to plan the sample size of new experiments through a power analysis.
• What is the additional value of these data? Additional value is given by the R syntax code that can be found in the supplemental material. This code allows to follow the meta-analysis step by step.

## 1 Data description

In the repository five files are available: *prisma.png, review.csv, data.csv, Supplemantal_Material.pdf,* and *Supplemental_Material.R.*

In *prisma.png* the PRISMA flow-chart is presented to summarize the stages of the literature search carried out to select eligible studies.

In *review.csv* the main characteristics of the selected studies are presented. This allows other researchers to easily assess the relevant information about the studies. The following variables are reported:

• *Author*: authors’ name.
• *Year*: year of publication.
• *Country*: country where the study was conducted.
• *N*: total sample size.
• *Ratio_M_F*: number of boys out of girls included in the study (when reported).
• *School_level*: school level of the participants (preschool, primary school, or high school).
• *Treatment:* type of program used in the intervention “*tutorial_practice*” for tutorial or drill & practice and “*video_game*” for videogames.
• *Treatment_description:* specific mathematical topic and software used in the intervention.
• *Sessions:* number of total sessions and length in week of the intervention.
• *Duration:* duration of each session.
• *Treatment_n:* sample size of the treatment group.
• *Control_group:* characteristics of the control group. Possible levels are “*passive*” if the control group did not receive any kind of intervention, “*active_math*” or “*active_spelling*” if the control group was trained respectively in mathematics (no digital-based interventions) or in a different topic such as spelling, and “*active_normative*” or “*passive_normative*” if the control group was formed by children without mathematical difficulties who participated or not to the intervention.
• *Control_n:* sample size of the control group.
• *Effects_measured:* specific outcomes measured to evaluate mathematical performance.
• *URL:* link to directly access the original paper.

In *data.csv* the data needed to compute the effect sizes of the selected studies are presented. Single studies can contribute with one or more effects. In the latter case, the type of dependence between effects within the same study is specified. The following variables are reported:

• *Id_study:* unique id to identify the study.
• *Author*: authors’ name.
• *Year*: year of publication.
• *Author_year*: author and year of publication to cite the study.
• *Treatment:* type of program used in the intervention “*tutorial_practice*” for tutorial or drill & practice and “*video_game*” for videogames.
• *Id_treatment:* id to identify different treatment groups within the same study.
• *Control_group:* characteristics of the control group. Possible levels are “*passive*” if the control group did not receive any kind of intervention, “*active_math*” or “*active_spelling*” if the control group was trained respectively in mathematics (no digital-based interventions) or in a different topic such as spelling, and “*active_normative*” or “*passive_normative*” if the control group was formed by children without mathematical difficulties who participated or not to the intervention.
• *Id_control:* id to identify different control groups within the same study.
• *School_level*: school level of the participants (preschool, primary school, or high school).
• *Outcome:* id to identify different outcomes measured within the same study.
• *N:* total sample size (treatment group and control group) for the effect considered.
• *Id_unique_effect:* unique id to identify the effect considered.
• *Id_effect:* id to identify the different effects within the same study.
• *Dependency:* type of dependency between effects reported in the same study. Possible levels “*none* ” if only one effect was coded for that study, “*multiple_outcomes*” if multiple outcomes were measured on the same participants, “*multiple_groups*” if the different treatment group were compared with the same control group, “multiple_outcomes_groups” if multiple outcomes were measured on the same participants and also different treatment group were compared with the same control group, and “*independent*” if outcomes were measured on independent participants in both treatment and control groups.
• *T_n:* number of participants in the treatment group.
• *T_m_pre:* treatment group mean performance in the pre-intervention conditions.
• *T_sd_pre:* treatment group standard deviation in the pre-intervention conditions.
• *T_m_post:* treatment group mean performance in the post-intervention conditions.
• *T_sd_post:* treatment group standard deviation in the post-intervention conditions.
• *C_n:* number of participants in the control group.
• *C_m_pre:* control group mean performance in the pre-intervention conditions.
• *C_sd_pre:* control group standard deviation in the pre-intervention conditions.
• *C_m_post:* control group mean performance in the post-intervention conditions.
• *C_sd_post:* control group standard deviation in the post-intervention conditions.
• *r_pre_post:* correlation between participants pre- post-intervention performance. Only one study reported the value of the correlation.
• *R_outcomes:* correlation between multiple outcomes. This is a relevant information when multiple outcomes are measured on the same participants but none of the study reported these correlations.
• *URL:* link to directly access the original paper.
• *Note:* further important information regarding the selected studies are listed. They specify if other outcomes, groups, or delayed post-intervention scores were reported in the original studies but not coded in the present file as not relevant to the aims of the present meta-analysis. However, other researchers might find these additional data useful. Notes concerns also participants, type of intervention and outcome characteristics. Moreover, it has been reported if specific outcomes have an inverse scoring (higher scores represent worse performances).

In *Supplmental_Material.pdf* all the steps of the meta-analysis are described and commented together with the R code syntax. In the first part, issues related to the effect size index used to summarize studies results and how to deal with the problem of multiple effects within the same study are discussed. Subsequently, each selected study is described and effects sizes are computed. Finally, the meta-analysis results together with the R code syntax are presented.

In *Supplemental_Material.R* plain R code syntax is reported to reproduce the meta-analysis results.

## 2 Experimental design, materials, and methods

Data were collected from studies included in the meta-analysis. The aim of the meta-analysis was to evaluate the effects of digital-based interventions for children with mathematical learning difficulties compared to control conditions in a group-designed randomized controlled trials.

To select primary studies a literature search was carried using the PRISMA protocol [2]. The literature search was conducted until March 2019 by means of PsycINFO, Google Scholar, and Educational Resources Information Center (ERIC) databases.

The following target terms were used to identify relevant studies: mathematics; dyscalculia; videogames; interventions; computer-assisted instruction; educational technology; mathematical learning; mathematics teaching; number sense; mathematics achievement; mathematical difficulties; randomized; controlled; control group; control condition.

Eligible studies were selected according the following inclusion criteria:

1 Studies had to evaluate the effects of digital-based interventions for children with mathematical learning difficulties in a group-designed randomized controlled trials.
2 Studies had to report students’ performance in any domain of mathematics as their dependent variable in the pre- and post-intervention conditions.
3 Studies could have been conducted in any country, but only English-language articles published in peer-reviewed journals were included.

Articles were screened for eligibility by three independent authors. After the screening 15 articles were included in the meta-analysis out of the 161 articles identified from the literature search.

Selected studies differed according to the type of program used in the intervention (based on videogames or on tutoring and drilling strategies), intervention characteristics (length and topic of the intervention), control group characteristics (active or passive control group) and school level of the participants (preschool, primary school, or high school). Selected studies evaluated children's performance in different domain of mathematics using their own set of questionnaires and tests. Moreover, selected studies used different experimental designs. Some studies evaluated multiple outcomes considering the same participants, other studies included different treatment and/or control groups, or they measured participants also in a delayed post-intervention condition.

For each study, only information relevant to the goals of meta-analysis was coded using a standardized form. Primary studies were coded taking into account the characteristics of the intervention (length, type, and topic of the intervention), the characteristics of the control group (passive or active control group), the outcomes measured to evaluate children's mathematical performance, school level of the participants, sample size of the treatment and control group, and mean and standard deviation of the mathematical performance in the pre- and post-intervention conditions. Below the 15 selected studies are briefly presented specifying which effects were included in the meta-analysis.

In Aunio and Mononen [3] two control groups were included. One control group is passive, whereas the other control group is an active control group trained in a topic other than math (i.e. spelling). In the meta-analysis only the treatment and the passive control groups were considered. Moreover, the study reports pre-test, immediate, and delayed post-tests scores on two different scales and on overall score. Only data collected at the pre-test and immediate post-test considering the overall scores were included in the meta-analysis.

In Baroody et al. [4] participants were evaluated on different mathematical tasks. In the meta-analysis only four mathematical tasks, which are related from a theoretical prospective, were considered.

In Baroody et al. [5] two treatment groups and one control group were evaluated on different mathematical tasks. In the meta-analysis only two mathematical tasks, which are related from a theoretical prospective, were included considering both treatment groups and the control group.

In Burns et al. [6] third- and fourth-grade students were evaluated separately. Thus, in the study there are two treatment groups and two control groups according to grade.

In Castro et al. [7] the standard deviations were not reported, but they were provided by the authors on request.

In Fuchs et al. [8] participants completed different mathematical and spelling tasks but in the meta-analysis only the performance in one mathematical task was considered.

In Hassler Hallstedt et al. [9] participants were evaluated on different mathematical tasks and the study included a passive control group, an active spelling control group, a math treatment group, and a math plus working memory treatment group. In the meta-analysis only four mathematical tasks, which are related from a theoretical prospective, were included considering the passive control group and the math treatment group. Note that the study actually reported the values of the standard errors, thus they have to be transformed in order to get the standard deviations values.

In Käser et al. [10] two groups were evaluated on different mathematical tasks in three different moments (T1, T2, and T3). Between T1 and T2 one group participated to the intervention and the other worked as a control group. Whereas, between T2 and T3 the treatment was offered to both groups. In the meta-analysis measures at T1 and T2 were included, considering only three mathematical tasks which are related from a theoretical prospective. In particular, the second task has an inverse scoring (higher scores represent worse performances). Thus, to compute the effect size pre- and post-intervention condition have to be inverted in order to get higher values of the effect size if children got an advantage from the intervention.

In Kucian et al. [11] treatment and control group were evaluated on several abilities but only one mathematical task was relevant to the meta-analysis. However, note that the control group is formed by children with age-appropriate calculation performance who participated to the intervention. Given these characteristics of the control group results of this study were not included in the final meta-analysis.

In Leh and Jitendra [12] treatment and control group were evaluated in three different moments: pre-test, immediate, and delayed post-tests. Only data collected at the pre-test and immediate post-test were included in the meta-analysis.

In Mohd Syah et al. [13] participants were evaluated on three different mathematical tasks and an overall score is provided. Only the overall was considered in the meta-analysis.

In Nelson et al. [14] one treatment group and two control groups were evaluated on two different mathematical outcomes. One control group is passive, whereas the other control group is an active control group trained in math. Only the treatment and the passive control groups were included in the meta-analysis considering one outcome of interest.

In Räsänen et al. [15] two treatment groups and one control group were evaluated on several outcomes. Only one outcome was relevant to the meta-analysis and it is a measure of reaction times. Thus, higher scores represent worse performances and to compute the effect size pre-post intervention condition have to be inverted in order to get higher values of the effect size if children got an advantage from the intervention. However, the control group was formed by children with age-appropriate calculation performance. Thus, study results were not be included in the final meta-analysis.

In Salminen et al. [16] treatment and control group were evaluated four times (T1, T2, T3, and T4) on four different mathematical tasks. Between T1 and T2, only the treatment group participated to the intervention whereas between T3 and T4 the treatment group received no extra intervention and the control group was offered another type of intervention. Only measures at T1 and T2 were considered in the meta-analysis.

In Stultz [17] overall scores for treatment and control group were reported.

Readers who want to use these data are highly recommended to refer to the original papers in order to check if study characteristics respect their own inclusion criteria.

## Declaration of Competing Interest

The authors declare that they have no known competing financial interests or personal relationships which have, or could be perceived to have, influenced the work reported in this article.
